# Emergence of *pstS*-Null Vancomycin-Resistant *Enterococcus faecium* Clone ST1478, Canada, 2013–2018

**DOI:** 10.3201/eid2609.201576

**Published:** 2020-09

**Authors:** Melissa McCracken, Robyn Mitchell, Stephanie Smith, Susy Hota, John Conly, Tim Du, John Embil, Lynn Johnston, Debbie Ormiston, Jennifer Parsonage, Andrew Simor, Alice Wong, George Golding, for the Canadian Nosocomial Infection Surveillance Program

**Affiliations:** Public Health Agency of Canada, Winnipeg, Manitoba, Canada (M. McCracken, T. Du, G. Golding);; Public Health Agency of Canada, Ottawa, Ontario, Canada (R. Mitchell);; University of Alberta, Edmonton, Alberta, Canada (S. Smith);; Toronto General Hospital, Toronto, Ontario, Canada (S. Hota);; University of Calgary Cumming School of Medicine, Calgary, Alberta, Canada (J. Conly);; Health Sciences Centre, Winnipeg (J. Embil, D. Ormiston);; Queen Elizabeth II Health Sciences Centre, Halifax, Nova Scotia, Canada (L. Johnston);; University of Alberta Hospital, Edmonton (J. Parsonage);; Sunnybrook Health Sciences Centre, Toronto (A. Simor);; Saskatchewan Health Authority, Saskatoon, Saskatchewan, Canada (A. Wong)

**Keywords:** emergence, antimicrobial resistance, enterococci, Enterococcus faecium, bacteria, vancomycin, *pstS*-null vancomycin-resistant clone, sequence type, ST1478, Canada

## Abstract

Rates of vancomycin-resistant enterococci bloodstream infections have remained relatively low in Canada. We recently observed an increase of 113% in these infections rates, which coincided with emergence of *Enterococcus faecium pstS*-null sequence type 1478. The proportion of this sequence type increased from 2.7% to 38.7% for all tested isolates from 2013–2018.

Vancomycin-resistant enterococci (VRE) are major nosocomial pathogens that have been observed worldwide (*1*,*2*). VRE were identified in Canada in 1993 (*3*), but rates of colonization and infection have remained relatively low for years (*4*,*5*). VRE bloodstream infections (BSIs) are of particular concern because they are associated with increased illness, length of hospital stay, healthcare costs, and death (*6*–*8*). Furthermore, increased rates of VRE BSI have been reported (*8*).

Since 1999, the Canadian Nosocomial Infection Surveillance Program (CNISP) has conducted surveillance of VRE BSIs, which includes collection of patient epidemiologic data and laboratory analysis of blood isolates, including multilocus sequence typing (MLST) and antimicrobial drug susceptibility testing (*5*,*9*). The MLST scheme for *Enterococcus faecium* relies on the sequences of 7 essential housekeeping genes (*10*). However, in recent years, MLST nontypeable strains of *E. faecium* have emerged that do not harbor the *pstS* gene (*11*). These *pstS*-null sequence types (e.g., sequence type [ST] 1421 and ST1424) are believed to have occurred through multiple inversion events and have been reported to be rapidly spreading in Australia, Denmark, and the United Kingdom (*11*–*13*). We report emergence and molecular characterization of a *pstS*-null sequence type (ST1478) that is rapidly disseminating across acute care hospitals in Canada.

## The Study

CNISP is administered by the Public Health Agency of Canada and has conducted prospective surveillance for VRE infection and colonization since 1999 (*5*,*9*). CNISP is a partnership between the Centre for Communicable Disease and Infection Control and the National Microbiology Laboratory at the Public Health Agency of Canada and sentinel hospitals that participate as members of the Canadian Hospital Epidemiology Committee, a subcommittee of the Association of Medical Microbiology and Infectious Diseases Canada. Hospitalized patients with enterococcal bacteremia characterized as having vancomycin MICs >8 mg/L were eligible (*9*). A patient was included more than once if a positive VRE blood isolate was identified >14 days after completion of therapy for a previous infection and believed to be unrelated to previous infection in accordance with best clinical judgement (*9*). Epidemiologic data were collected and VRE BSI isolates were forwarded to the National Microbiology Laboratory for further characterization (*9*).

All isolates that failed to give a sequence type by conventional MLST (*10*) were subjected to whole-genome sequencing on the MiSeq Platform (Illumina, https://www.illumina.com). Assembled reads (contigs) were analyzed by using an in-house MLST tool based on 1 from the Center for Genomic Epidemiology website (https://cge.cbs.dtu.dk/services).

During 2013–2018, a total of 797 VRE BSI cases were reported among 62 participating acute care hospitals across 10 provinces for which 608 VRE BSI isolates were submitted. During this surveillance period, the rate of VRE BSI significantly increased from 0.16 cases/10,000 patient-days to 0.34 cases/10,000 patient-days (p<0.001) (Figure). These rates are much higher than those reported during 1999–2009 (0.005 cases/1,000 admissions during 1999 to 0.068 cases/1,000 admissions during 2009) (*5*). Of the 608 VRE BSI collected, different MLST types were identified, which included 4 of the newly reported *pstS-*null sequence types ST1478 (n = 115), ST1421 (n = 7), ST1424 (n = 2), and ST1612 (n = 1). The increase of ST1478 isolates from 2.7% in 2013 to 38.7% in 2018 coincides with the increase in VRE BSI rates (Figure). After emergence of ST1478, STs that once dominated (ST18, ST117, and ST412) dramatically decreased. This shift in clonal types is similar to what has been reported in other countries after identification of these *pstS-*null mutants (*12*).

**Figure:**
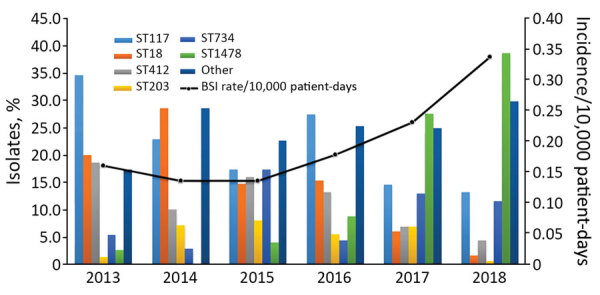
Increase in annual vancoymicin-resistant enterococci BSI rates and emergence of novel *pstS*-null sequence type ST1478, Canada, 2013–2018. BSI, bloodstream infection; ST, sequence type.

As of 2018, ST1478 has been identified in 19 of 62 CNISP participating hospitals in 6 provinces. Of the 19 hospitals, 12 were from western Canada (British Columbia, Alberta, Saskatchewan, and Manitoba), 6 from central Canada (Ontario and Quebec), and 1 from eastern Canada (Newfoundland and Labrador, Prince Edward Island, New Brunswick, and Nova Scotia). Regionally, ST1478 represented 23.7% (65/274) of isolates tested from western Canada, 14.8% (49/332) of isolates tested from central Canada, and 50% (1/2) of isolates tested from eastern Canada during 2013–2018. Furthermore, among the VRE BSI identified in western Canada, a significantly higher proportion were ST1478 (56.5%, 65/115) compared with non-ST1478 (42.4%, 209/493; p = 0.006).

The predominant *van* gene among ST1478 isolates was *vanA* (99.1%, n = 114); only 1 isolate harbored a *vanB* gene (0.9%). We determined resistance to antimicrobial drugs by using broth microdilution and GPALL1F Sensititer panels (Trek Diagnostics, http://www.trekds.com). We interpreted MICs by using breakpoints described by the Clinical and Laboratory Standards Institute (*14*). Antimicrobial drug susceptibility tests showed that ST1478 isolates have increased resistance to chloramphenicol (10.4%), daptomycin (13.0%), HL-gentamicin (80.0%), and tetracycline (91.3%) compared with non-ST1478 isolates (Table 1). We verified daptomycin nonsusceptibility by using Etest (bioMérieux, https://www.biomerieux.com). Clinically, increased daptomycin nonsusceptibility among the ST1478 isolates is of particular concern because daptomycin is 1 of the few remaining treatment options for VRE and resistance to it is an increasing clinical problem (*15*). CNISP surveillance data show a nonsignificant increase in the use of daptomycin to treat patients with VRE bloodstream infections. During 2015, a total of 53.7% of patients with a VRE BSI were given daptomycin, and during 2018, this proportion increased to 61.1% (p = 0.5).

**Table 1:** Antimicrobial drug resistance results for ST1478 versus non-ST1478 isolates of vancoymicin-resistant enterococci, Canada, 2013–2018*

Antimicrobial drug	% ST1478, n = 115	% Non-ST1478, n = 493	p value
Ampicillin	100.0	100.0	1
Chloramphenicol	10.4	1	0.0001
Ciprofloxacin	100.0	100.0	1
Daptomycin	13.0	3.9	0.0014
Erythromycin	100.0	92.1	0.6068
HL gentamicin	80.0	13.6	0.0001
Levfloxacin	100.0	99.6	1
Linezolid	1.7	0.4	0.1669
Nitrofurantoin	16.5	35.9	0.002
Penicillin	100.0	100.0	1
Quinupristin/dalfopristin	0.9	8.5	0.0032
Rifampin	95.7	90.1	0.7104
HL streptomycin	15.7	39.8	0.0002
Tetracycline	91.3	46.0	0.0001
Tigecycline	0.9	0.4	0.4776
Vancomycin	100.0	97.2	0.8835

*HL, high level; ST, sequence type.

During 2013–2018, we identified 115 ST1478 VRE BSI isolates among 110 patients. The median age of ST1478 patients was 58 years (interquartile range 52–69 years), and most (63.1%, 69/111) were male. The most commonly identified risk factors at the time of positive culture included use of a central venous catheter (69.5%, 57/82), solid organ transplant recipient (24.7%, 24/97), receiving hemodialysis (21.4%, 15/70), and receiving chemotherapy (15.7%, 11/70). A total of 31% (28/92) of ST1478 patients were already in an intensive care unit at the time of positive culture, and an additional 11 (12.1%) were admitted within 30 days of positive blood culture. The all cause 30-day mortality rate was 32.4%. Bacteremia patients with ST1478 VRE were similar with respect to age, sex, intensive care unit admission, and mortality rate compared with patients who had non-ST1478 VRE. Patients with ST1478 VRE were more likely to have undergone solid organ transplantation (24.7%) than patients with non-ST1478 VRE (12.9%; p = 0.005) (Table 2). However a VRE outbreak was reported by 1 center in their multiorgan transplant unit, which might explain this finding.

**Table 2:** Characteristics for patients with ST1478 versus non-ST1478 vancoymicin-resistant enterococci bloodstream infections, Canada, 2013–2018*

Characteristic	ST1478, n = 110	Non-ST1478, n = 492	p value
Mean age, y (SD)	58 (16.8)	59 (17.5)	0.89
Sex			
M	69/110 (62.7)	296/492 (60.2)	0.62
F	41/110 (73.3)	196/492 (39.8)	0.62
Central venous catheter	57/82 (69.5)	184/251 (73.3)	0.50
Solid organ transplant	24/97 (24.7)	42/325 (12.9)	0.005
Hemodialysis	15/70 (21.4)	47/223 (21.1)	0.95
Chemotherapy	11/70 (15.7)	54/223 (24.2)	0.14
ICU admission within 30 d of positive blood culture	11/91 (12.1)	60/332 (18.1)	0.18
30 d all-cause mortality rate	35/108 (32.4)	120/390 (30.8)	0.75

*Values are no. positive/no. tested (%) unless indicated otherwise. ICU, intensive care unit.

## Conclusions

The emergence of the *pstS-*null mutant ST1478 identified in acute care hospitals in Canada coincides with a major increase in VRE BSI rates. This increase might be attributed to an increased virulence/fitness of this strain type or changes in VRE infection prevention and control practices.

Clinically, the increased proportion of daptomycin nonsusceptibility among this emerging strain type is of concern. Future work, including whole-genome sequencing, and collection of enhanced epidemiologic and infection prevention and control practices data are being undertaken to provide a better understanding of the transmission, clonal relatedness, and evolution of this strain type within and between hospitals across Canada. Clinicians should be aware of these drug-resistant bacteria.
